# Reactive granulomatous dermatitis as a manifestation of primary vitreoretinal lymphoma

**DOI:** 10.1016/j.jdcr.2025.12.041

**Published:** 2026-01-02

**Authors:** Emilio Bert-Mangino, R. Angélica Méndez-Pérez, Marcela Saeb-Lima, Linda García-Hidalgo

**Affiliations:** a Department of Dermatology, Instituto Nacional de Ciencias Médicas y Nutrición Salvador Zubirán, Mexico City, Mexico; b Department of Pathology, Instituto Nacional de Ciencias Médicas y Nutrición Salvador Zubirán, Mexico City, Mexico

**Keywords:** medical dermatology, reactive granulomatous dermatitis, primary vitreoretinal lymphoma, palisaded neutrophilic and granulomatous dermatitis

## Introduction

Reactive granulomatous dermatitis (RGD) comprises a spectrum of dermatoses, including palisaded neutrophilic and granulomatous dermatitis (PNGD).^1^^,^^2^ Up to 77% of RGD cases are associated with systemic diseases, most commonly autoimmune conditions such as rheumatoid arthritis and systemic lupus erythematosus. Nevertheless, a non-negligible proportion involve haematologic malignancies, as illustrated in this case.^3^^,^^4^

We report the case of a 71-year-old woman with RGD, PNGD pattern, which ultimately led to the very rare diagnosis of a primary vitreoretinal B-cell lymphoma. This case underscores the importance of recognizing RGD as a dermatological marker of systemic malignancies, enabling timely diagnosis and intervention.

## Case report

A 71-year-old woman was referred to our institution in April 2023 with suspected sarcoidosis. She had a history of bilateral panuveitis and a prior diagnosis of “disseminated annular granuloma” in 2019 based on an external skin biopsy. She had been taking prednisone (5 mg daily), hydroxychloroquine (200 mg daily), and methotrexate (7.5 mg weekly).

**Fig 1:**
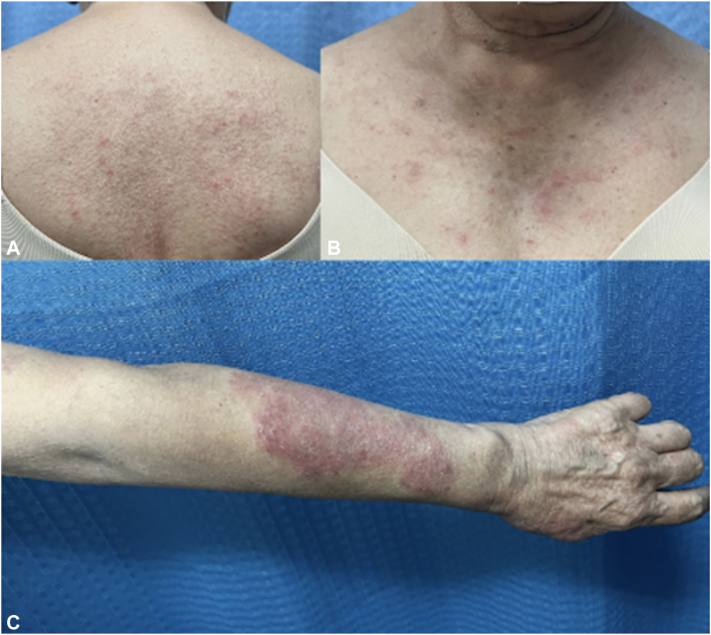
Initial clinical presentation of reactive granulomatous dermatitis **(A-C)**.

**Fig 2:**
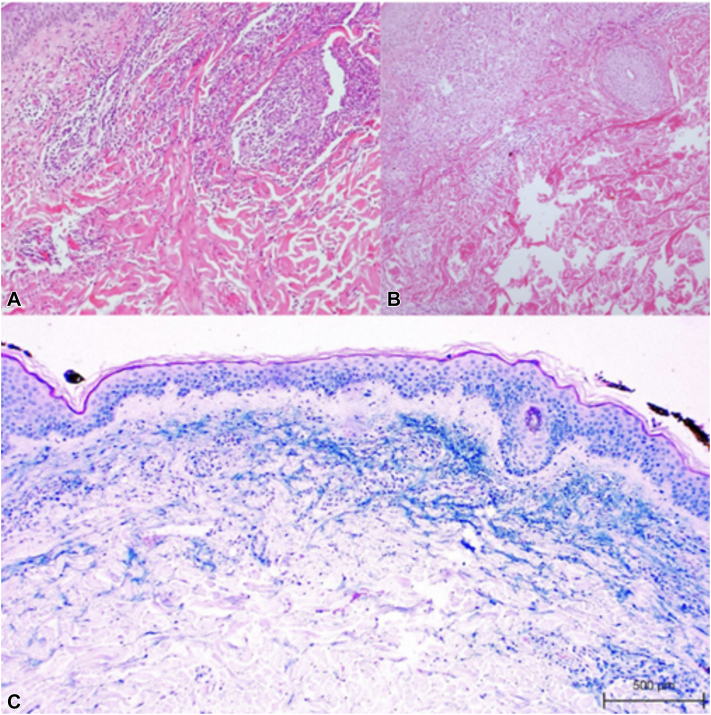
Punch biopsy, **(A)** showing palisaded granulomatous inflammation surrounded by numerous neutrophils. Histiocytes surround thickened and degenerating collagen **(B)** with interstitial mucin deposition **(C)**. (HE, 40×).

Her initial evaluation in our department occurred in June 2023. Clinical examination revealed erythematous-oedematous plaques, some with a semi-annular configuration on the trunk and extremities, associated with pruritus (Fig 1, *A*-*C*). The dermatosis had previously achieved apparent remission in December 2022 after initial treatment, followed by recurrence in 2023, associated with worsening ocular symptoms. As part of her evaluation, a 4-mm punch biopsy was performed. The biopsy revealed a palisaded neutrophilic and granulomatous pattern (Fig 2, *A* and *B*). Mucin was present but limited (Fig 2, *C*), supporting a diagnosis within the RGD spectrum rather than granuloma annulare.

Laboratory findings showed antinuclear antibodies (IgG HEp-2-IFI) with a coarse speckled and mitochondrial pattern at 1:640 titre and lymphopenia (430 cells/μL). Other autoimmune markers as anti-dsDNA, anti-Ro/SSA, anti-La/SSB, anti-Smith, and anti-RNP antibodies, along with complement levels (C3, C4), viral serologies, angiotensin-converting enzyme and QuantiFERON-TB Gold were negative.

During evaluation, the patient developed headache, fever and progressive bilateral vision loss. Extensive assessment was performed to rule out systemic neurological involvement. Cerebrospinal fluid analysis revealing mild pleocytosis (118 mg/dL) and the magnetic resonance imaging (MRI) demonstrated thickening of the lens and ciliary body in the right eye. Positron emission tomography-computed tomography (PET-CT) showed increased metabolism in the right palpebral region. Given these ophthalmological findings, she underwent phacoemulsification and vitrectomy. Histopathology was suggestive of marginal zone B-cell lymphoma, confirmed by the presence of the p.L265P mutation in the MYD88 gene, establishing a diagnosis of primary vitreoretinal B-cell lymphoma without systemic involvement.

**Fig 3:**
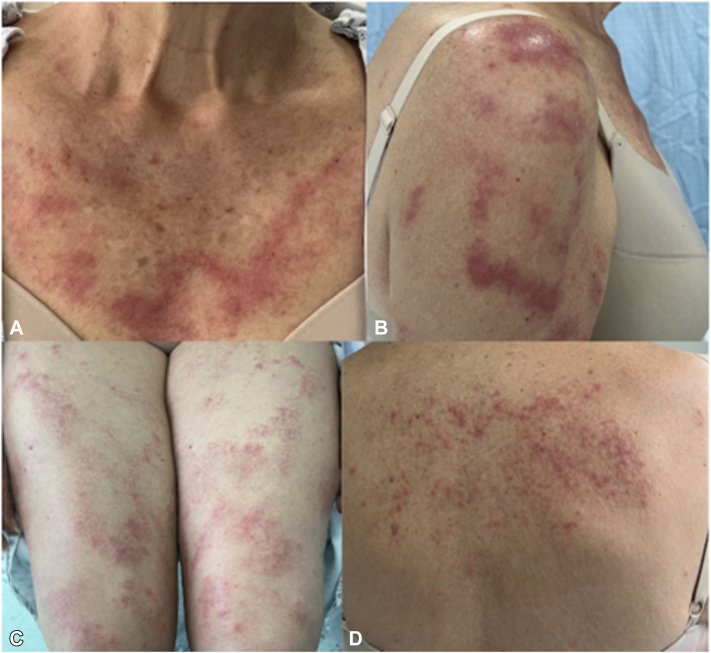
Recurrence of dermatosis associated with persistent lymphoma **(A-D)**.

Intravitreal rituximab was initiated, resulting in a partial response of the lymphoma. Consequently, treatment was escalated to systemic chemotherapy, achieving a favourable response and remission of the dermatosis. However, in June 2024, recurrence of the dermatosis was documented (Fig 3), and simultaneously, follow-up PET-CT revealed persistent activity of the underlying lymphoma.

## Discussion

RGD encompasses a spectrum of 3 histopathological patterns: PNGD, interstitial granulomatous dermatitis, and interstitial granulomatous drug reactions including interstitial granulomatous drug reaction (IGDR).^1^^,^^2^ The presence of interstitial mucin deposition, highlighted by Alcian blue staining, can aid in differentiating RGD from granuloma annulare, as the latter typically exhibits more abundant mucin within the areas of collagen degeneration.

Often emerging as a cutaneous response to systemic disease, up to 77% of patients have an associated condition, with 80% identified before or at dermatosis onset. Notably, in a considerable proportion of cases, the associated disease is identified after the initial presentation of the dermatosis, highlighting the importance of long-term surveillance in patients without an identifiable systemic disorder at baseline.^3^

Rheumatologic disorders constitute the majority of underlying conditions (52.3%), with rheumatoid arthritis and systemic lupus erythematosus accounting for 10.8% and 7.7% of cases, respectively, followed by haematologic malignancies including acute myeloid leukaemia (AML), multiple myeloma, and Hodgkin lymphoma.^3^

A recent systematic review including 458 cases of RGD reported malignancies in 12.5% of patients, with haematological neoplasms accounting for 74.1%. Among these, myelodysplastic syndromes (18.6%), chronic myelomonocytic leukaemia (14.8%), non-Hodgkin lymphoma (14.8%), and AML (11.1%) were the most frequent, followed by Hodgkin lymphoma (7.4%), multiple myeloma (7.4%), and mycosis fungoides (3.7%).^5^

There appears to be a consistent pattern in the timing of haematologic malignancy diagnosis relative to dermatosis onset. In the Bangalore Kumar et al cohort, 3 of 5 patients (60%) had a prior concomitant diagnosis, whereas in 2 patients (40%) the malignancy was identified after the appearance of skin lesions, similar to our patient. Likewise, in the larger review by Yang et al, 7 cases (26%) were detected during follow-up.

Given the potential severity and prognostic implications of delayed oncological diagnosis, maintaining a high index of clinical suspicion and performing a symptom-driven systemic evaluation are essential. In patients with RGD, investigation should be tailored to suspected associations with autoimmune diseases, malignancies, infections, or other systemic conditions.^1^ As demonstrated in our patient, this strategy allowed identification of the associated systemic entity.

In conclusion, this case underscores that reactive granulomatous dermatitis should be regarded not as a diagnosis per se, but as a cutaneous manifestation of systemic conditions; thus, control of the underlying disease is essential to control the dermatosis.

## Conflicts of interest

None declared.
